# What stops Korean immigrants from accessing child and adolescent mental health services?

**DOI:** 10.1186/s13034-022-00455-0

**Published:** 2022-03-03

**Authors:** Chohye Park, Jik H. Loy, Steven Lillis, David B. Menkes

**Affiliations:** 1 Waikato District Health Board, Hamilton, New Zealand; 2 University of Auckland, Auckland, New Zealand; 3 University of Auckland, Waikato Clinical Campus, Hamilton, New Zealand

**Keywords:** Korean immigrant, New Zealand, Child and adolescent, Mental health service access, Stigma, Barriers to healthcare

## Abstract

**Background:**

Access to child and adolescent mental health services by ethnic minorities has been poorly studied. Despite rapid growth of the immigrant Korean population, evidence indicates that few Korean families utilise these services in New Zealand. Those that do tend to present late and with significant morbidity. We sought to understand barriers to service access from Korean parents’ perspectives.

**Method:**

Seven focus groups were undertaken with 31 Korean parents of children aged 18 and under. The focus groups were semi-structured, held in the Korean language and utilised two case scenarios of common childhood/adolescent mental illnesses around which a set of broad, open-ended questions were posed. All conversations were audiorecorded, transcribed and translated into English. Thematic analysis was conducted using NVivo software.

**Results:**

Both attitudinal and structural barriers were identified. Attitudinal barriers included attribution of mental illness to external stressors or parenting problems, social stigma, denial or normalization of children’s behaviour, fear of family disempowerment, and mistrust of public mental health services. Structural barriers included parents’ lack of information regarding available services, logistical difficulties in access, communication difficulties, concerns over the quality of translators, and cultural competence of service providers.

**Conclusion:**

Significant barriers prevent Korean immigrant families from accessing child and adolescent mental health services in New Zealand. Measures to improve access, for example by countering stigma, are urgently required.

**Supplementary Information:**

The online version contains supplementary material available at 10.1186/s13034-022-00455-0.

## Introduction

Korean immigration to both Australia and New Zealand (NZ) is relatively recent compared with other Asian ethnic groups. Since 1990, immigration has increased markedly and Koreans now constitute the 4th largest Asian ethnicity in NZ [1]. Taken together, Asians comprise more than 12% of population and are now the second largest ethnic minority, after Maori [2]. In the United States and Canada, low access of mental health services by Asian immigrants led to an assumption that prevalence rates of mental illness were low, but this has not been borne out by epidemiological studies which show rates similar to the mainstream population [3]. Asian patients tend to present with more severe and chronic mental illness than those of patients from other cultural backgrounds and consequently require more intensive treatment and longer hospitalization [4].

There is little epidemiologic data specifically focused on Asian children in NZ, Australia or the United States, but some evidence indicates that Asians, like other youth in NZ, report relatively high levels of depression, suicidal thoughts and suicide attempts [5]. Similarly, Korean epidemiological studies indicate that rates of adolescent depression and ADHD in that country are similar to those in the West [6]. Suicide rates among adolescent Koreans are the highest among countries of the Organisation for Economic Cooperation and Development (OECD) and the 2nd highest cause of adolescent mortality [7].

Utilisation of child and adolescent mental health services in NZ is even lower for Asians than for Maori or Pacific, other ethnic minorities known to have reduced access [8]. This trend is particularly notable in the North Island of NZ where the rate of attendance of Asian youth to child and adolescent mental health services outpatient clinics is one tenth that of Maori. However, inpatient admission rates during the same period were high, suggesting late presentation and comparable rates of serious disorder [8].

In light of obvious unmet need, and the evidence that early intervention is associated with improved outcome, failure of minority groups to access mental health services is a serious concern. In this study we explore reasons for the low rates of access to child and adolescent mental health services by the Korean community in NZ.

## Method

Focus groups were used to examine Korean parents’ attitudes and knowledge. Women are mainly responsible for child rearing and therefore it is common to find families where the father lives in Korea and the remainder of the family lives in New Zealand. Hence, our focus was on the experiences and beliefs of Korean women. However, the opportunity arose to also undertake one focus group of Korean fathers living in NZ. We decided not to mix male and female participants because Korean females tend to defer to males and this would be expected to affect group dynamics and the quality of the discussion. Two common clinical scenarios, childhood attention deficit hyperactivity disorder (ADHD) and adolescent depression, were chosen and presented to each group, followed by a set of semi-structured questions for the participants to discuss, all in the Korean language. The semi-structured interview format in Korean and English is presented in Additional files 1 and 2.

Potential participants were contacted through the Korean community via notices in churches, schools, and Korean community groups in Hamilton, NZ. A total of 31 participants were recruited of which 4 were male. All participants were parents with children aged between 4 and 18 years, all but one had a university qualification, and most were between 35 and 55 years. Written informed consent was obtained from all participants; no remuneration was offered. Seven focus groups of 4–6 participants were facilitated by the first author, conducted during the evening in an informal setting (the home of one of the participants), each lasting between 2 and 3 h. Each session was audiorecorded, transcribed and translated by the first author.

Thematic analysis [9] was used to identify, analyse and report themes in the data. A coding system was developed through discussion of the transcripts by all four authors. The thematic analysis used text segments identified and coded into assigned categories. These were grouped into possible themes and, with re-reading and refinement, major themes developed; results from male (n = 1) and female (n = 6) focus groups were similar and pooled for analysis. The software package NVivo 8 (QSR International) was used to organise and structure transcript analysis. Results from all authors were integrated to increase coding reliability.

## Results

We identified several, partly overlapping, factors that prevented access to child and adolescent mental health services; two major themes emerged: attitudinal and structural.

### Attitudinal barriers

1. Attribution of children’s emotional or behaviour problems to external stressors and/or parenting deficits
  Focus group participants tended to attribute problems to absent parents, parenting deficits, or alternatively as the consequence of external stress, such as school difficulties, racism, migration stresses, or puberty. External attribution of the child’s emotional and behavioural problems also led to Korean parents feeling guilty.
  “You need both parents to bring up the children; if his father is away in Korea then it is not going to be easy for his mother to control this child’s behaviour...”
  “I do not consider this case to be depression. I believe that this is problem with puberty and would try to deal with it, or simply just wait for him to grow up. I would not believe any serious things would happen to my son. I see this as a temporary misfit with school and would consider changing the environment...”
  “I would feel bad and guilty about my child’s problems: I would look at my behaviour and interaction with my child to see whether I could change the situation...”
2. Stigma
  With a scenario indicating an illness requiring mental health service involvement, social stigma and a sense of shame was a major concern.
  “Going to the mental health service is a huge stigma. Just telling others that I went to psychiatric hospital would be a very stressful situation. I would not want anyone to know about my son seeing a psychiatrist. I would not even talk to my husband about going to Child and Adolescent Mental Health Services even if I had to go...”
  “I reveal my weakness (as a parent) and I worry about other people’s opinion of me because I know we (Koreans) are good at judging others...”
  “The first image coming to my mind about mental illness is about seeing an iron bar. For us (Koreans), mental illness means craziness and it carries huge stigma and shame beyond just having an illness... In Korea, child psychiatric clinics are called ‘children’s behavioural clinic’ instead...”
3. Denial/normalization of children and young person’s behavioural and emotional problem
  Participants tended to normalise the behavioural and emotional problems presented in the scenarios.
  “Well at that age, I thought about suicide many times, I think it is normal to feel like that at least once at this age. They will usually grow out of this”
  “This boy sounds just like any other boy I know, boys have to be active and curious..”
4. Fear of family disempowerment leading to service avoidance and attempting to manage by themselves
  Korean parents expressed fear of unknown services and were concerned over losing control of their child’s care. The strong emotional response to the idea of referral to child and adolescent mental health services seemed to be related to concern over the unknown as well as fear that their parental rights and opinions would not be respected.
  “I would feel very offended by a suggestion of the school counsellor to take my child to Child and Adolescent Mental Health Services for assessment. I would change school rather than go for an assessment. I know my child better than the school counsellor and mental health professionals would come to their own conclusions about my child’s problem which I have no influence over... even if this was a mental illness, I would like to find another way to manage it...”
  “They might take my son and put him into a psychiatric hospital which I do not want to happen...”
  “If I have done all I could do, such as talking to my child and trying to make sense of the situation... I would go on the internet, talking to people who live overseas (in a similar situation). I will also reflect on my time as a teenager and think though trying to understand my son’s behavioural changes. If all these things do not work, then I will ask my parents because they have had experience of raising me...”
5. General mistrust of Western mainstream systems, particularly regarding cultural competency
  Even if the Korean parents choose to attend child and adolescent mental health services, they were sceptical about the capacity of the service to understand underlying cultural issues.
  “New Zealanders have different way of understanding and looking at children’s issues..."
  “Their understanding of our culture is minimal.., my daughter was distressed because she could not keep up with her peer group at high school; most of them were busy with boyfriend and sexual issues instead of focusing on study for the future...”
  “Korea has better health care system with high calibre doctors and you could choose anyone you would like to see for your children’s problem...”
  “Their understanding of our hopes, dreams and expectation we have for our children is minimal and their way of bringing up children is very different... New Zealand youth are given too much freedom and choice...”
  “I do not think they understand the Korean way of looking at the issues; how many of them understand Hwa Byung (a culture bound syndrome with elements of somatisation) [10]? In Korea, we know without asking questions what this is about, how would they understand the sacrifice parents make for the next generation and maintain the family name...”
6. Seeking help in Korean churches, or from Korean counsellors or traditional doctors, or returning to Korea to seek help in Korean mental health services
  If Korean parents decided to access help for their children’s behavioural or emotional problems, their first choice of contact was someone in the Korean community they believed had the necessary authority and experience. If necessary, many indicated they would take their children back to Korea for help from various sources—including Korean child mental health services and child psychiatrists.
  “Instead of seeing mental health professionals, I would seek help from a counsellor who can speak Korean to talk about my child’s problem... they are good at listening and give us good suggestions...”
  “I would ask my church pastor who is experienced with human emotional problems...”
  “At least I can talk to Korean doctors in Korean, we can discuss things in same language...”
  “I will take my child to Korea to get a better assessment because we have the same understanding about social rules, cues and nuances with regards to our children’s behaviour...”

### Structural barriers

1. Lack of information about child and adolescent mental health service and marked difference in psychiatric services
  None of our 31 participants were aware of the existence of public child and adolescent mental health services or relevant non-governmental organisations. This lack of understanding appears to arise from clear differences between Korean and New Zealand medical systems.
  “The medical systems are so different. In NZ, there is no choice and we have to wait to go through the public system... I do not know any private specialists... There is little choice for us. While in Korea, we can choose who treat our child. We can see as many doctors as we like in order to confirm or dispute the professional opinions. Instead of parents deciding about our child, we depend on the professionals’ opinion...I have no say in my child’s condition and treatment in NZ.”
  “Until I met you [the first author] I did not know that this country provides child and adolescent mental health service for free. My GP never advised me about it when I discussed my child’s problem, they do not provide any information about the available services...”
2. Logistic difficulties in accessing the service
  “Even if I did know the service existed, I do not know how to contact them... the medical system in NZ is so different from Korea where we can go and see any doctor we want... You have to rely on your GP to make decisions... ”
  “I do not know where the child and adolescent mental health team works. Are they in the hospital? Who should I contact in order to get help? If I ring someone and they started talking in English, I do not think I have the guts to ask for help”
3. Language and communication difficulties in using a translator, notably because of differences in language structure and emotional expressions.
  “I have many experiences with using translators in the hospital service, most of them are no better than me in translating the pertinent issues in my story, they cut short my story and over simplified the issues. I had a friend who developed appendicitis and I knew this was the case... but the translator conveyed it was not serious until it ruptured and became peritonitis; they had to operate on her...The translator made me so angry and upset...”
  “I do not believe the translator can deliver the exact meaning of feelings I express... I worry about losing the meaning in translation. In child and young person’s emotional problems, translation is so important and I do not think the translators working in these areas are up to the task. I have little confidence in their capacity”.
  “I have to rely on my children to communicate with mainstream service providers... this causes problems in communication. I understand English better than I can say it... despite having higher qualifications in Korea, we all have difficulties in communicating in spoken English...”

## Discussion

The relevant literature is limited, even with regard to difficulties faced by Koreans in accessing child and adolescent mental health services in their own country. One survey identified a lack of information about available services, prejudice about mental illness, and misconceptions regarding severity being the main obstacles to accessing services [11]. Our results show similar problems as well as additional difficulties related to immigration from a traditional Asian culture to a Western, English-speaking country.

Our analysis identified two groups of barriers, attitudinal and structural/systemic. The most prominent attitudinal barriers included perceived shame, embarrassment and guilt in having children with emotional and/or behavioural problems. These were generally considered a parental responsibility and helps explain why parents avoid service referral and attempt to solve problems by themselves or within their own immigrant community.

Shame plays a key role in social control and underlies the enforcement of rules in Confucius philosophy-influenced Asian countries, including Korea [12]. The shame of losing face for the individual is shared by the wider family and community itself. Accordingly, the disturbed behaviour that accompanies mental illness tends to cause shame and embarrassment in the family and wider community. As a result, mental illness is often kept hidden and secret.

Despite being given the explanation that our two scenarios reflected treatable mental illness, instead of experiencing relief from what might be seen as a face-saving alternative, most parents objected to this explanation and actively disputed the illness model. This attitude appears related to both mental health literacy and stigma. Thus, when stigma is perceived as too high a price to pay, parents disregard illness as a model for explaining children’s behavioural and emotional problems.

In Korea, both mental and physical illnesses were traditionally attributed to superstitious belief systems until contact with Western concepts following the Korean War in 1950. After this, and subsequent rapid industrialisation, belief systems regarding mental illness have evolved. Koreans have been integrating Western models of both physical and mental illness. Child and adolescent mental health is no longer a taboo subject in Korea and is now commonly discussed in the media, including television programs such as “Live Good Morning” that include discussion of common cases by mental health professionals.

In our focus group discussions, there was very little attribution of mental illness to superstitious or ancestral curses, unlike findings among Chinese immigrants to Australia [13]. This difference may relate, in part, to the high educational attainment of the Korean parents in our sample.

There has been a misconception based on cultural stereotypes that Asians are model citizens and therefore experience lower rates of mental illness. However this hypothesis has been challenged, and it pointed out that Asians with mental illness often suffer in silence until the family and community are unable to cope, leading to delayed and ambivalent engagement with mainstream service providers [14].

Korean immigrants who have been less integrated into their host culture tend to adhere to beliefs in attitudes prevalent at the time they left Korea. Such individuals, as parents, appear to avoid contact with mental health services for their children. It was evident in focus groups that those with experience of child mental health services in Korea were more positive toward such services in NZ. Additionally, recent immigrants or parents of international students appeared to show better levels of mental health literacy compared to Korean immigrants who had lived in NZ for longer.

Structural barriers included lack of information about service providers and how to access them, and concerns regarding service providers’ cultural competency, specifically regarding non-Koreans’ ability to understand the nuances and underlying meaning of the family problems. Remarkably, none of our participants was aware regional child and adolescent mental health services or how to access these. Likewise, they were unaware of the Health and Disability Code of Patient Rights and the free provision of translators.

In addition to language barriers, differences in mental health service organisation may add to difficulties in access. For example, Korea has universal mandatory public health insurance, and citizens can seek the opinion of any doctor of their choice. Specialist or hospital based services do not require formal referral, and healthcare access and choice are thus very much consumer based. Our findings suggest that this systemic difference added to Korean immigrant parents’ fear of losing control in managing mental disorder in their children.

The majority of our participants preferred opting out of public mental health services in favour of alternatives, such as seeking help through family networks and community organisations, notably Korean churches. Most agreed that they would, if necessary, go back to Korea to seek an opinion from a Korean child psychiatrist or mental health service. Our findings thus emphasise the importance of culture and language barriers and also the perceived lack of accessible information regarding mental health services.

In 2002 the NZ Mental Health Commission recommended promoting mental health in Asian communities by increasing public support for cultural diversity, providing information, English language education, developing community support programmes and increasing service providers’ awareness of Asian cultural issues [8]. Unfortunately, after 15 years there is little evidence to indicate effective nationwide implementation of these recommendations or indeed changes in the way Asian populations tend to view and access mental health services. Thus, although these results were derived from one region (Waikato), they are likely to reflect more general barriers to clinical service access in NZ.

On the other hand, some regional initiatives are relevant, including a Korean mental health and addiction awareness group (Like Minds, Like Mine, 2016). Asian mental health support groups have also developed in Auckland (Independent Living Services, 2013), New Zealand’s largest city and one with a proportionately higher Asian population than elsewhere in the country. Similarly, the Mental Health Foundation, a charitable trust, has developed Kai Xin Xing Dong, a public education program to reduce stigma for Asian youth experiencing mental illness (Mental Health Foundation, 2017). The impact of these initiatives is as yet uncertain.

## Limitations

Our study explored parental attitudes regarding help-seeking on behalf of children; it did not address attitudes of either parents or children regarding their own mental health. Despite overlaps with other Asian populations, Korean culture has many distinctive features, potentially limiting the generalisability of our results. Similarly, our sample of Korean parents in NZ had notably high educational attainment, likely related to NZ immigration criteria, and so our results may also have been affected by this factor.

Participating parents were recruited largely through the first author’s Korean school and church involvement. Most participated with an expressed willingness to help Korean families in NZ, but their knowledge of the first author may have influenced responses. At the time of interview, no parents were involved with child and adolescent mental health services. However, after one focus group, a mother approached the first author regarding her teenaged daughter’s disordered eating, later seeking help from Korea.

## Conclusion

Both attitudinal and structural barriers prevent adequate mental health service delivery to the Korean community in NZ. To overcome these barriers, a more concerted effort is required beyond provision of translators for clinical encounters. The importance of education and information dissemination among minority communities is essential, as is developing the appropriate cultural sensitivity and competency of mental health staff. The concept of cultural competency in mental health service providers has gained traction with evidence of improved access and better engagement in treatment [15, 16].

Shortly after conclusion of the project, the first author presented a summary of results to an evening meeting of the local Korean community. Based on these findings, the local child and adolescent mental health service has made several changes, including more proactive provision of translators, added emphasis on confidentiality, closer links to high school guidance counsellors, and education of staff regarding Korean and other minority cultures and their implications for service access.

## Supplementary Information

**Additional file 1.** Semi-structured interview (Korean).

**Additional file 2.** Semi-structured interview (English) both cited in Method.

## Data Availability

Participant information sheets, consent forms, scenario details, and de-identified transcripts are available on reasonable request from the corresponding author.

## Abbreviations

ADHD: Attention deficit hyperactivity disorder
NZ: New Zealand
OECD: Organisation for Economic Cooperation and Development
