# Supplementary material for: What stops Korean immigrants from accessing child and adolescent mental health services?
Source: Child Adolesc Psychiatry Ment Health. 2022 Mar 3;16:19. doi: 10.1186/s13034-022-00455-0 (PMC8895579; doi:10.1186/s13034-022-00455-0)
Supplement: Supplementary file 1 — Additional file 1. Semi-structured interview (Korean). [file 13034_2022_455_MOESM1_ESM.docx]

Attachment two (English version)

Scenario one

He is a 7-year-old Korean boy, who showed hyperactivity, inattention, and poor impulse control since he started walking. His parents were repeatedly called by the kindergarten teachers due to behavioral problems with the other children; he tended to disrupt play and insisted on doing things his own way. His primary school teachers expressed their concerns about his hyperactivity, difficulties in concentration, talkativeness, inattention, interrupting other students, and difficulty taking turns. His parents reported that he is consistently unable to finish his homework and is restless and fidgety when they read a book together. He is forgetful and always loses his belongings. He needs his mother to organize him in the morning for school, packing his bag and reminding him of his daily routines. When he goes to the Korean church, he climbs on the table and climbs the trees. He does not seem to learn from his mistakes and keeps making the same mistakes. He does not respond well when given consequences for his bad behaviors. Despite being intelligent, his school achievement is below average, and his teachers are worried about his learning achievement. He likes outdoor activities and running around rather than sitting still to read. His parents are professionals working in NZ and have good reputation in the local community. They do not have marriage problems or difficulties with anger. His temperament is different from his siblings. His parents are exhausted with managing his difficult behavior and meeting his many demands.

Questions for the participants

1. What is wrong with him and what has caused his problems?
2. If this happens with your child, what would you do?
3. If his school suggests a referral to the child and adolescent mental health service for assessment of possible ADHD, what would you do?
4. Would you accept a referral or not? why would you make this choice?
5. What are your alternatives?
6. How would you to deal with this problem?
7. Are you able to get any other support and, if so, how would you go about it?
8. Are you aware of any services in this city which might provide support?

Scenario two

He is a 14-year-old student, who started his first-year in high school 6 months ago. His family noticed he had become irritable, angry, prone to crying and “giving up”. His school performance has been poor, and his school results have been going downhill. He started fighting with other children, which is out of character. He told his parents he has been bullied at school. He has lost about 3kg in weight over a month and stopped eating. He has difficulty sleeping and feels tired all the time. He complains about headaches. He is forgetful and has poor concentration. He feels helpless and hopeless. He feels his life is a failure. His parents met his schoolteachers who said they wanted to help. However, he did not think anyone is helping. He says life is not worthwhile and he wants to be dead.

Questions for the participants

1. What is wrong with this young person and what is the cause of his problem?
2. If this happens with your child, what would you do?
3. If his schoolteachers and counsellor suggest a referral to Child and Adolescent Mental Health Services to assess for depression, what would you do?
4. Would you consent to this referral or not? why would you make this choice?
5. What does the Child and Adolescent Mental Health Service do?
6. What do you think your rights as a parent are when your child is being assessed by Child and Adolescent Mental Health Services?
7. Do you have any alternative explanations for this boy’s problems?
8. How would you manage this problem?
9. Are you able to get alternative support and, if so, how would you go about it?
10. Are you aware of any services in this city which might provide support?
