# Supplementary material for: What stops Korean immigrants from accessing child and adolescent mental health services?
Source: Child Adolesc Psychiatry Ment Health. 2022 Mar 3;16:19. doi: 10.1186/s13034-022-00455-0 (PMC8895579; doi:10.1186/s13034-022-00455-0)
Supplement: Supplementary file 2 — Additional file 2. Semi-structured interview (English) both cited in Method. [file 13034_2022_455_MOESM2_ESM.doc]

시나리오 1.

만 7살인 한국 남자아이는 두살때 부터 항상 주의력이 산만하고 행동이 지나치게 과잉적합니다. 아이는 유치원다니기 시작하면서 부모님들은 선생님께 자주불려 다녀읍니다. 지금 국민학교에서도 문제가 많아 부모님들은 선생님께 자주불려다녀읍니다. 집에서 학교숙제를 하면서 집중하는데 큰 어려움을 가지고 있고 부모님의 말을 잘 안 들 때가 많습니다. 어떤때는 질문이 끝나기도 전에 참지 못하고 대답하고 남의 대화나 놀이를 가로 막는 경우가 많습니다. 아이는 차례를 기다리는데 힘이 들고 말을 지나치게 많이 합니다. 매일 매일의 활동을 잘 기억하지 못하고 잊어버리는 경우가 많은데 아이가 학교에서 문제를 일으키지 않게 엄마가 모든 일이 해야 할 정도입니다. 아이는 자주 물건을 잃어버리고 주위 것들에 의해 쉽게 주의가 산만해 집니다. 다른 친구를 방문하거나 교회에 가서 가구나 나무등에 올라가거나 주위를 뛰어다닙니다. 한번은 지붕위에 올라가 놀고있었던적도 있읍니다. 아이는 충동적으로 행동을 자주하고 난중에 어떤결과가 생기는가 생각을 잘하지 않하는경우가 많습니다. 참을성이없어 화가 나면 다른아이을 때릴때도 있읍니다. 영리한아이인데도 학교에서 성적이 좋지않읍니다. 아이는 책읽기나 단어공부는 좋아하지않고 밖에서 뛰어노는것을좋아 합니다. 아이의 가족은 한국인 교민 사회에서 잘 적응했으며 존경받고 있습니다. 하지만 아이의 부모들은 이제아이문제로 지칠때가 많은겄 처럼 보입니다. 아이는 다른 형제들에 비해 유난히 성격이 다른것같읍니다.

참가자를 위한 질문

1. 이 아이에게 무엇이 일어나고 있다고 생각하십니까?
2. 여러분의 가족에게 일어난 일이라면 어떻게 하시겠습니까?
3. 만약 학교 선생님이/ 가정의가 아이에게 아동 및 청소년 정신과 진료 를 받기를 권유한다면 어떻게 하시겠습니까?
4. 여러분께서 이 진료 받겠읍니까? 안 받겠읍니까? 각각의 이유는 무엇입니까?
5. 여러분께 다른 선택은 무엇입니까?
6. 여러분은 어떻게 처리하겠습니까?
7. 여러분은 어떤 방법으로 필요한 자료/지원을 얻을 수 있습니까?
8. 여러분께서는 도움을 줄 수 있는 해밀턴의 다른 기관들에 대해 아십니까?

시나리오 2.

만 14세인 한국남자 아이는 6개월 전 고등학교로 옮기고 나서 급격히 변했습니다. 아이는 짜증과 화를 잘 냅니다. 좌절했을 때는 가끔 울기도 합니다. 자신 본연의 모습이 전혀 아니며 학교 성적은 계속 떨어집니다. 학교에서 문제를 일으킵니다. 아이는 학교에서 괴롭힘을 당한다고 호소하고 싸움에 말려 듭니다. 먹는 것도 점점 줄어들고 지난 달동안 적어도 3kg정도 살이 빠졌습니다. 잠을 제대로 잘 수 없으며 아침에 매우 피곤해 합니다. 자주 두통을 호소하며 잊어버리게 되고 학교공부에 집중할 수 없다고 말합니다. 아이는 자신에게 미래는 없다고 말하면서 자신을 실패자라고 생각합니다. 아이의 부모님은 학교 선생님을 만나 아이가 학교에서 괴롭힘을 받는것에 대해 이야기했습니다. 교사는 아이를 돕기로 약속했습니다. 그러나 아이는 여전히 과롭힘을 당한다고 느끼고 싸움을 계속 하게 되면서 학교는 아이의 공격적인 행동을 이유로 정학을 고려하기에 이릅니다. 아이는 누구도 자기를 상관하지 않으며 도울 수 없다고 말합니다. 자기에겐 미래도 없고 죽고 싶다는 말을 하기도 합니다.

참가자를 위한 질문

1. 이 아이에게 어떤 일이 벌어지고 있다고 생각하십니까?

2. 여러분의 가족에게 일어난 일이라면 어떻게 하시겠습니까?

3. 만약 학교 선생님이/가정의가 아이에게 아동 및 청소년 정신과 진료를 받기를

권유한다면 어떻게 하시겠습니까?

4. 여러분께서 이 진료 받겠읍니까? 안 받겠읍니까? 각각의 이유는 무엇입니까?

5. 여러분생각에 아동 및 청소년 정신과 진료소에서 어떤일을 하는지요?

6. 여러분이 아동 및 청소년 정신과 진료소에서 부모로서 어떤 권리가 있는지 아십니까?

7. 여러분께 가질 수 있는 다른 선택은 무엇입니까?

8. 여러분은 어떻게 처리하겠습니까?

1. 여러분은 어떤 방법으로 필요한 자료/지원을 얻을 수 있습니까
2. 여러분께서는 도움을 줄 수 있는 해밀턴의 다른 기관들에 대해 아십니까?
